# T cell receptor sequencing identifies prior SARS-CoV-2 infection and correlates with neutralizing antibodies and disease severity

**DOI:** 10.1172/jci.insight.150070

**Published:** 2022-05-23

**Authors:** Rebecca Elyanow, Thomas M. Snyder, Sudeb C. Dalai, Rachel M. Gittelman, Jim Boonyaratanakornkit, Anna Wald, Stacy Selke, Mark H. Wener, Chihiro Morishima, Alexander L. Greninger, Michael Gale, Tien-Ying Hsiang, Lichen Jing, Michael R. Holbrook, Ian M. Kaplan, H. Jabran Zahid, Damon H. May, Jonathan M. Carlson, Lance Baldo, Thomas Manley, Harlan S. Robins, David M. Koelle

**Affiliations:** 1 Adaptive Biotechnologies, Seattle, Washington, USA.; 2 Stanford University School of Medicine, Stanford, California, USA.; 3 Department of Medicine, University of Washington, Seattle, Washington, USA.; 4 Fred Hutchinson Cancer Research Center, Seattle, Washington, USA.; 5 Department of Epidemiology,; 6 Department of Laboratory Medicine and Pathology,; 7 Department of Immunology,; 8 Department of Microbiology, and; 9 Department of Global Health, University of Washington, Seattle, Washington, USA.; 10 National Institute of Allergy and Infectious Diseases (NIAID) Integrated Research Facility, Frederick, Maryland, USA.; 11 Microsoft Research, Redmond, Washington, USA.; 12 Benaroya Research Institute, Seattle, Washington, USA.

**Keywords:** COVID-19, Infectious disease, Adaptive immunity, Diagnostics, T cell receptor

## Abstract

**BACKGROUND:**

Measuring the immune response to SARS-CoV-2 enables assessment of past infection and protective immunity. SARS-CoV-2 infection induces humoral and T cell responses, but these responses vary with disease severity and individual characteristics.

**METHODS:**

A T cell receptor (TCR) immunosequencing assay was conducted using small-volume blood samples from 302 individuals recovered from COVID-19. Correlations between the magnitude of the T cell response and neutralizing antibody (nAb) titers or indicators of disease severity were evaluated. Sensitivity of T cell testing was assessed and compared with serologic testing.

**RESULTS:**

SARS-CoV-2–specific T cell responses were significantly correlated with nAb titers and clinical indicators of disease severity, including hospitalization, fever, and difficulty breathing. Despite modest declines in depth and breadth of T cell responses during convalescence, high sensitivity was observed until at least 6 months after infection, with overall sensitivity ~5% greater than serology tests for identifying prior SARS-CoV-2 infection. Improved performance of T cell testing was most apparent in recovered, nonhospitalized individuals sampled > 150 days after initial illness, suggesting greater sensitivity than serology at later time points and in individuals with less severe disease. T cell testing identified SARS-CoV-2 infection in 68% (55 of 81) of samples with undetectable nAb titers (<1:40) and in 37% (13 of 35) of samples classified as negative by 3 antibody assays.

**CONCLUSION:**

These results support TCR-based testing as a scalable, reliable measure of past SARS-CoV-2 infection with clinical value beyond serology.

**TRIAL REGISTRATION:**

Specimens were accrued under trial NCT04338360 accessible at clinicaltrials.gov.

**FUNDING:**

This work was funded by Adaptive Biotechnologies, Frederick National Laboratory for Cancer Research, NIAID, Fred Hutchinson Joel Meyers Endowment, Fast Grants, and American Society for Transplantation and Cell Therapy.

## Graphical abstract

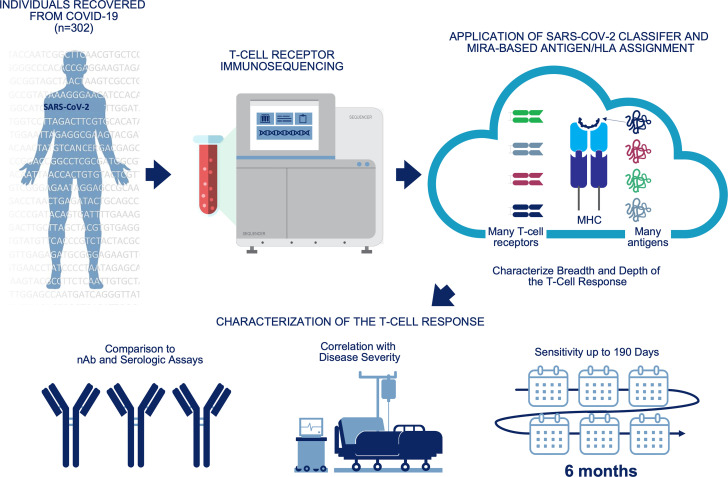

## Introduction

Understanding the immune response to SARS-CoV-2 is essential to inform clinical management and vaccination strategies for COVID-19 (1). SARS-CoV-2 infection induces both humoral and T cell responses, but the nature and kinetics of these responses vary with disease severity and individual characteristics (2–4). Antibody titer and T cell assays have demonstrated that SARS-CoV-2 vaccines induce humoral and/or cell-mediated immune responses, but the optimal combination of responses underlying immune correlates of protection remains undefined (5, 6). This knowledge gap is underscored by recently described viral variants that can escape antibody responses (7, 8) but maintain largely preserved CD4^+^ and CD8^+^ T cell responses (9), potentially affecting vaccine-induced immunity and viral neutralization (10, 11).

While serologic assays are a common means of assessing prior SARS-CoV-2 infection at the population level (4, 12), whether serology results correspond with long-term protective immunity remains unclear (13, 14). Neutralizing antibody (nAb) titers, despite providing a measure of immune protection in SARS-CoV-2 infection (15), are challenging to assay, pose biohazard risks, and may have limited persistence (16).

More recently, T cell receptor (TCR) repertoire–based assays have emerged as another technology for reliable assessment of prior infection and immunity that can be performed using as little as 1–2 mL of whole blood (17, 18). Due to the extreme diversity of complementarity-determining region 3 (CDR3) sequences, most TCRs (~99%) are private, or unique, to any given individual. However, a subset of TCRs can be detected across multiple individuals, more commonly among those with shared HLA alleles; these public TCRs may be the result of exposure to a common antigen and can serve as a biomarker of disease (19, 20). We have developed classifiers leveraging thousands of public TCR sequences shared across individuals with a history of infection for identification of prior CMV infection and Lyme disease, in addition to prior SARS-CoV-2 infection (17, 21, 22). These classifiers are highly specific for the disease of interest; for example, the initial SARS-CoV-2 classifier was developed to have a target specificity of 99.8% across 1702 prepandemic controls, presumably including individuals exposed to other coronaviruses (17).

Previously, we used the SARS-CoV-2 TCR classifier to characterize the magnitude and kinetics of the T cell response after SARS-CoV-2 infection, documenting that the T cell response to SARS-CoV-2 peaks 1–2 weeks after infection and remains detectable for months after recovery (17). Analysis of blood samples from 70 individuals with PCR-confirmed SARS-CoV-2 infection showed that the magnitude of the T cell response is greater in those with symptomatic disease compared with asymptomatic disease and greatest in those who were hospitalized due to COVID-19 (23).

In the present study, in order to better characterize the T cell response to SARS-CoV-2 and assess the advantages and limitations of TCR testing relative to other modalities, we have analyzed blood samples from a prior study collected up to 6 months after symptom onset as part of a convalescent plasma donor screening program (24). The previous study compared the performance of 2 SARS-CoV-2 IgG serology tests relative to nAb titers, showing that both tests correlated well with nAb testing, enabling better prioritization of high-titer samples for Ig donor products. Here, we have conducted further assessments of samples from this cohort and additional enrolled individuals to characterize the T cell response to SARS-CoV-2 and correlations with antibody testing strategies and clinical indicators of disease. To better understand the advantages and limitations of TCR testing for assessing immunity to SARS-CoV-2, we leveraged the SARS-CoV-2 classifier to characterize the relative number of unique SARS-CoV-2–associated TCRs, defined as clonal breadth, and the level of expansion of SARS-CoV-2–associated TCRs, defined as clonal depth. We then examined the correlation between SARS-CoV-2–associated TCR depth or breadth and nAb titers. To develop a mechanistic understanding of the T cell response to SARS-CoV-2, we have used multiplex identification of antigen-specific TCRs (MIRA; ref. 25) to assign SARS-CoV-2–associated TCRs to specific viral antigens and class I or II HLA restrictions. We then conducted subanalyses to assess the correlations of these subpopulations with disease severity. We also compared the sensitivity of TCR testing relative to serologic assays in hospitalized and nonhospitalized individuals up to 190 days after symptom onset. Results of this study contribute to our understanding of the T cell response to SARS-CoV-2 and underscore the importance of understanding T cell–mediated immunity in addition to serologic immunity for informing the response to the COVID-19 pandemic.

## Results

### Participant demographics.

Individuals with a history of laboratory-confirmed SARS-CoV-2 infection who volunteered to be considered for convalescent plasma donation were recruited to this study. The characteristics of the initial 250 people in the cohort were described previously (24). As a result of ongoing recruitment, the present report extends this cohort to 302 people. Participant demographics are summarized in Table 1. For 55 of 302 individuals, an additional sample from a subsequent visit was available (median, 90 days between samples) for a total of 357 samples analyzed in this study.

### SARS-CoV-2–specific T cell responses were correlated with nAb titers.

**Figure 1:**
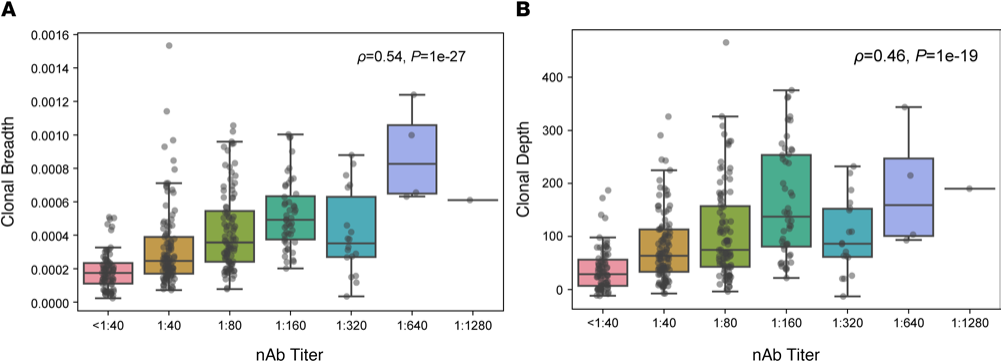
SARS-CoV-2–specific T cell responses correlate with nAb titers. (**A** and **B**) Correlation of T cell clonal breadth (**A**) and depth (**B**) with nAb titers in samples from individuals with a prior positive SARS-CoV-2 RT-PCR test (*n* = 357). Significance was evaluated by Spearman’s rank-order correlation. Data are expressed as median ± IQR.

To evaluate the association between SARS-CoV-2–specific T cell responses and nAb titers, TCR-β sequencing was performed on samples collected during convalescence (29–190 days after symptom onset; median, 80 days). We then evaluated the association between clonal breadth and depth of the T cell response and nAb titers. Clonal breadth was defined as the relative number of distinct SARS-CoV-2–associated T cell clonotypes as a fraction of the overall repertoire, and clonal depth was defined as the extent of expansion of SARS-CoV-2–associated T cells, as previously described (17). Both clonal breadth and depth showed significant positive correlations with nAb titers (Figure 1, A and B), suggesting that, like nAb titers (15), SARS-CoV-2 T cell response signatures may provide insight into protective immunity arising from natural infection.

**Figure 2:**
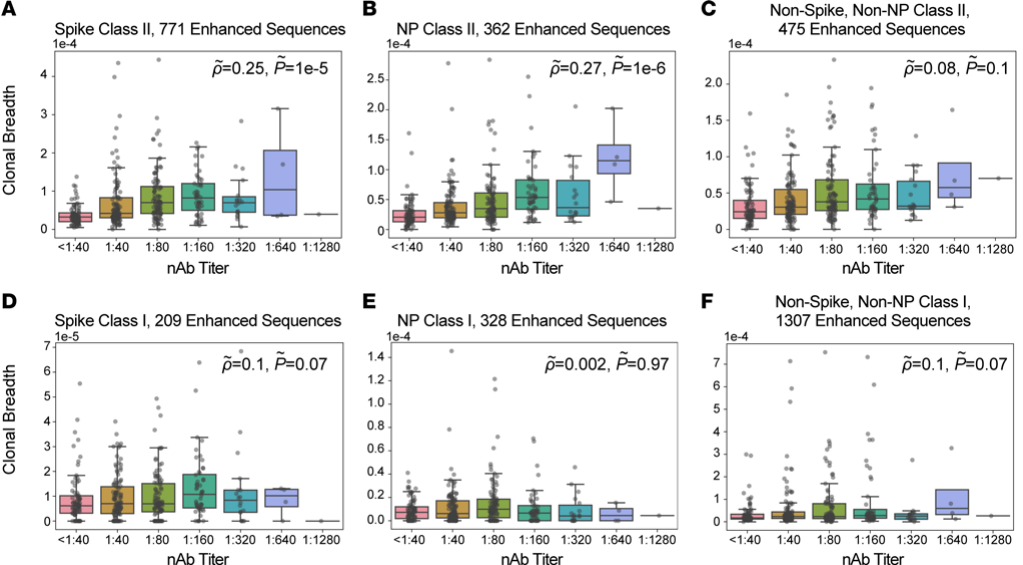
Correlation of TCR clonal breadth with nAb titers. (**A**–**F**) Class II HLA–restricted TCRs (**A**–**C**) or class I HLA–restricted TCRs (**D**–**F**) were assigned to antigens from the SARS-CoV-2 spike protein (**A** and **D**), nucleocapsid phosphoprotein (NP; **B** and **E**), and other viral proteins (**C** and **F**), and clonal breadth of assigned sequences was correlated with nAb titers. Correlations for spike class II–associated T cells (**A**); NP class II–associated T cells (**B**); other class II T cells (**C**); spike class I–associated T cells (**D**); NP class I–associated T cells (**E**); and other class I T cells (**F**) with nAb titer. Data are expressed as median ± IQR (*n* = 357). Partial correlations were assessed by Spearman’s rank-order correlation.

**Figure 3:**
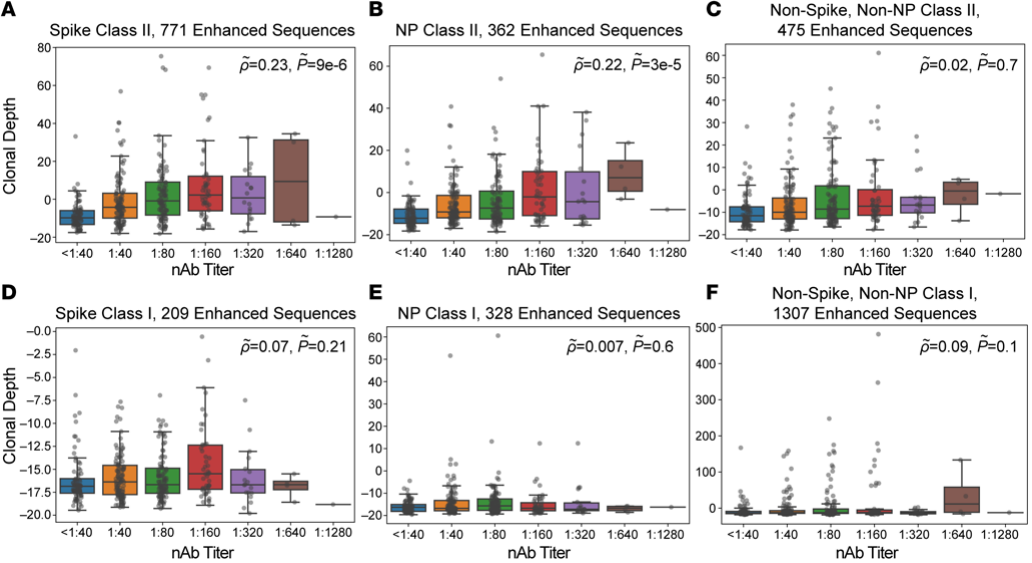
Correlation of TCR clonal depth with nAb titers. Class II HLA–restricted TCRs (**A**–**C**) or class I HLA–restricted TCRs (**D**–**F**) were assigned to antigens from the SARS-CoV-2 spike protein (**A** and **D**), nucleocapsid phosphoprotein (NP; **B** and **E**), and other viral proteins (**C** and **F**), and clonal depth of assigned sequences was correlated with nAb titers. Correlations for spike class II–associated T cells (**A**); NP class II–associated T cells (**B**); other class II T cells (**C**); spike class I–associated T cells (**D**); NP class I–associated T cells (**E**); and other class I T cells (**F**) with nAb titer. Data are expressed as median ± IQR (*n* = 357). Partial correlations were assessed by Spearman’s rank-order correlation.

To further characterize the correlation between T cell responses and nAb titers, we assigned a subset of TCR sequences to specific antigens and class I or class II HLA restriction based on data from multiplexed antigen-stimulation assays (17, 25). These TCRs are associated with cytotoxic (CD8^+^) and helper (CD4^+^) cellular immune responses, respectively. For CD4^+^ T cells, we assigned 769 TCRs to antigens from SARS-CoV-2 spike protein, 362 to nucleocapsid phosphoprotein, and 474 to other viral proteins. We then evaluated correlations between nAb titers and assigned TCR sets; partial correlations were applied to account for confounding correlations arising from natural biologic variation in the immune response to different antigens (see Methods). These analyses revealed that the clonal breadth and depth of class II–associated TCRs for spike protein and nucleocapsid phosphoprotein, but not other antigens, were correlated with nAb titer after partial correlation (Figure 2, A–C, and Figure 3, A–C). TCRs assigned to CD8^+^ T cells were not significantly correlated with nAb titers (Figure 2, D–F, and Figure 3, D–F), suggesting that the CD4^+^ T cell response is the primary origin of the nAb correlation with overall SARS-CoV-2–specific TCR breadth and depth.

To determine whether SARS-CoV-2–associated TCRs used in our analyses are associated with functional CD4^+^ T cell responses, we enriched CD4^+^ T cells from PBMCs collected from 3 people in our convalescent cohort who were hospitalized and admitted to intensive care for treatment of COVID-19. After recovery, samples were collected at 2 time points each. Whole SARS-CoV-2 virus was used as the stimulating antigen. Characterization of the TCR repertoires of SARS-CoV-2–stimulated cells compared with control PBMCs showed that activation-induced marker–sorted (AIM-sorted) CD4^+^ T cells were significantly enriched for members of our set of 4287 SARS-CoV-2–specific TCR clonotypes (or enhanced sequences; ref. 17), with 81 unique TCRs overlapping the enhanced sequence set out of a total of 8709 unique TCRs. This corresponded to a greater than 10-fold increase in clonal breadth of SARS-CoV-2–specific TCRs for ex vivo AIM-sorted CD4^+^ T cells compared with matched convalescent PBMCs (Supplemental Figure 1A; supplemental material available online with this article; https://doi.org/10.1172/jci.insight.150070DS1). We also noted considerable enrichment of our MIRA TCR set, with 41 unique TCRs overlapping the enhanced sequence set and the MIRA TCR set (Supplemental Figure 1B). These data suggest that the TCRs used in our analyses represent functional TCRs capable of responding to whole SARS-CoV-2 viral antigen after processing by antigen-presenting cells, although additional validation studies are required to confirm the functional status of all TCRs in the classifier.

### SARS-CoV-2–specific T cell responses were correlated with clinical measures of COVID-19 severity.

**Figure 4:**
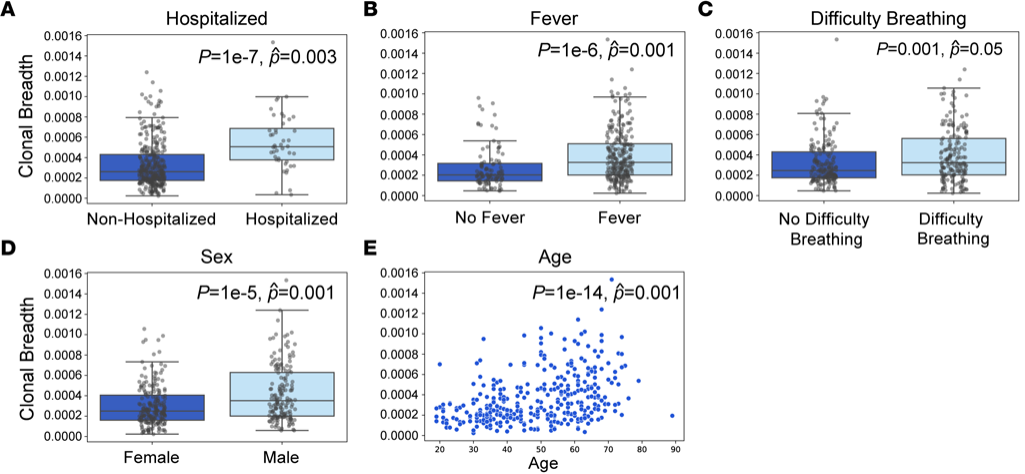
Association of T cell clonal breadth with clinical variables. (**A**–**E**) Correlation of clonal breadth with hospitalization (**A**), fever (**B**), difficulty breathing (**C**), sex (**D**), and age (**E**) was evaluated by univariate Mann-Whitney *U* test (*P*) and multivariate linear regression with age, sex, hospitalization, fever, difficulty breathing, and TCR rearrangements as variables. Data are expressed as median ± IQR (*n* = 302 convalescent individuals). Population means and 95% CI values for (**A**–**D**) were as follows: (**A**) nonhospitalized, 3.4 × 10^–4^ (3.1 × 10^–4^ to 3.7 × 10^–4^); hospitalized, 6.3 × 10^–4^ (5.1 × 10^–4^ to 7.5 × 10^–4^); (**B**) no fever, 2.8 × 10^–4^ (2.3 × 10^–4^ to 3.2 × 10^–4^); fever, 4.0 × 10^–4^ (3.7 × 10^–4^ to 4.4 × 10^–4^); (**C**) no difficulty breathing, 3.3 × 10^–4^ (3.0 × 10^–4^ to 3.6 × 10^–4^); difficulty breathing, 4.0 × 10^–4^ (3.6 × 10^–4^ to 4.4 × 10^–4^); (**D**) female, 3.1 × 10^–4^ (3.0 × 10^–4^ to 3.4 × 10^–4^); male, 4.4 × 10^–4^ (3.9 × 10^–4^ to 4.8 × 10^–4^).

Previous analyses involving this cohort revealed significant associations between nAb response and important clinical correlates, including older age, male sex, fever, difficulty breathing, and hospitalization (24). Similarly, clonal breadth of the SARS-CoV-2 T cell response was significantly correlated with each of these variables in both single and multivariable regression (Figure 4). Clonal depth was significantly correlated with all variables except difficulty breathing (Supplemental Figure 2). These associations were independent of the number of unique productive T cell rearrangements based on multivariable regression. Consistent with increased rates of severe illness and hospitalization observed in men and older adults (26), T cell breadth and depth were also higher in these groups (Figure 4, D and E, and Supplemental Figure 2, D and E).

### SARS-CoV-2–specific T cell responses had greater diagnostic sensitivity than serology for identifying past infection, particularly in nonhospitalized cases.

**Figure 5:**
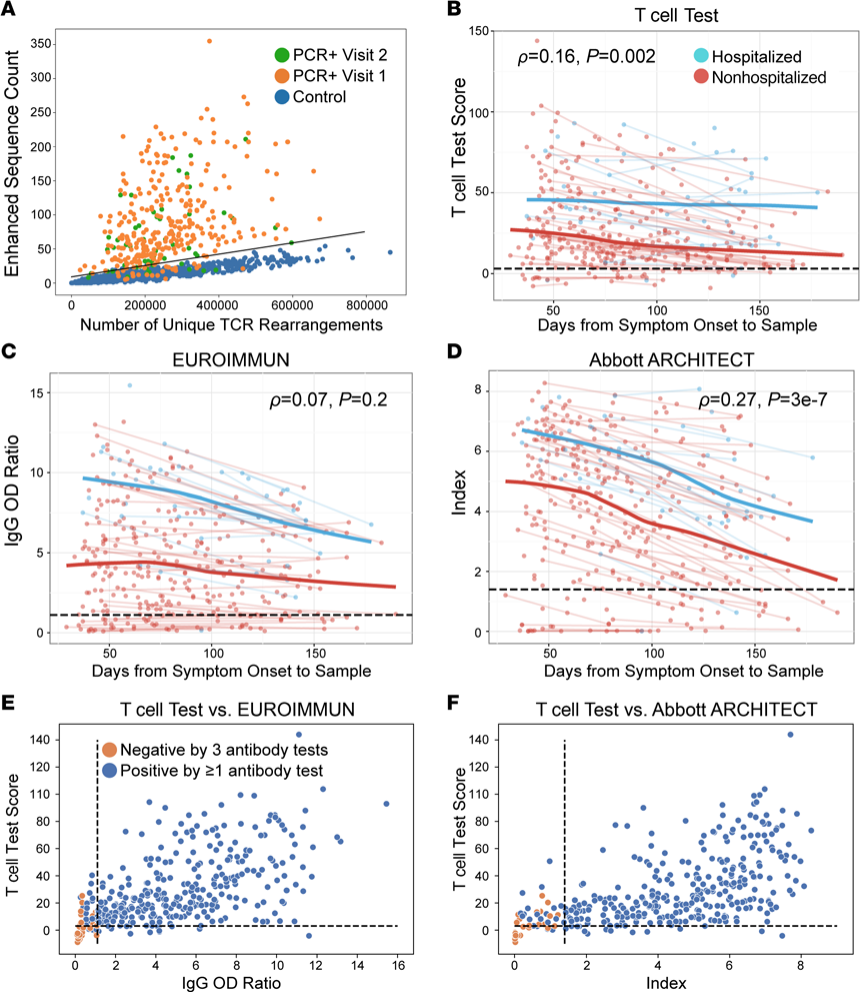
Comparison of TCR-based assay and serological assays. (**A**) Number of SARS-CoV-2 enhanced sequences (of 4287 possible) verus number of unique TCR rearrangements for RT-PCR–confirmed samples collected at visit 1 (*n* = 302; orange) or visit 2 (*n* = 55; green) or control samples (*n* = 1657; blue). Line indicates the classifier threshold. (**B**–**D**) T cell test score, defined as log-odds of the probability of the logistic regression model (**B**), EUROIMMUN IgG OD ratio (**C**), and Abbott ARCHITECT assay index (**D**) as a function of time from symptom onset for hospitalized (blue) or nonhospitalized (red) individuals for 357 samples from 302 convalescent individuals. Trend lines connect visit 1 and visit 2 samples from the same individual. Blue and red bold trend lines indicate smoothed means (locally estimated scatterplot smoothing, LOESS; ref. 56) for hospitalized and nonhospitalized individuals. Significance was evaluated by Spearman’s rank-order correlation. (**E** and **F**) T cell test scores versus EUROIMMUN IgG OD ratio (**E**) or Abbott ARCHITECT index (**F**) for 357 samples from 302 convalescent individuals. Samples classified negative by all 3 antibody tests (EUROIMMUN, Abbott ARCHITECT, nAb titer) are highlighted in orange. Black dashed lines indicate cutoffs for positivity/negativity. The cutoff for nAb positivity was 1:40 (24).

Based on identification of public SARS-CoV-2–specific T cell–enhanced sequence signatures shared across individuals, a classifier was developed to diagnose recent and past SARS-CoV-2 infection (17) and was validated in several independent data sets (18, 23). Optimization and application of this TCR classifier as a test for past SARS-CoV-2 infection yielded a sensitivity of 88.8% across all samples and time points (Table 2 and Supplemental Table 1), with a specificity of 99.8% using a control set of 1657 prepandemic samples (Figure 5A). Consistent with the observation that TCR repertoire breadth and depth are greater in individuals with severe disease (Figure 4, A–C, and Supplemental Figure 2, A–C; ref. 23), sensitivity of the classifier was higher in hospitalized (93.2%) versus nonhospitalized cases (88.2%; Supplemental Table 2). Although classifier scores decreased slightly with time from symptom onset, the assay maintained a sensitivity of 95% for samples collected > 150 days after onset (*n* = 20; Figure 5B and Table 2). Together, these results support the utility of the TCR-based assay as a sensitive measure of prior disease.

Next, we compared results from the TCR-based assay with 2 serology assays. The EUROIMMUN assay measures IgG binding to the S1 domain of the SARS-CoV-2 spike protein, and the Abbott ARCHITECT assay measures IgG binding to the nucleocapsid protein. Across the entire cohort and inclusive of all time points, sensitivity of these assays was significantly lower than TCR-based testing (Table 2 and Supplemental Table 1; ref. 24). Notably, when performance was assessed at specific sampling time points in the months following initial illness, both serology assays showed steeper declines in quantitative diagnostic scores over time relative to the TCR-based assay (Figure 5, C and D), with the greatest differences in sensitivity observed > 5 months (150 days) from initial symptoms (Table 2 and Supplemental Table 1).

We next explored the performance of TCR and serology tests among hospitalized and nonhospitalized individuals (Table 2 and Supplemental Table 2). All 3 tests exhibited high sensitivity (≥93.2%) for identifying prior SARS-CoV-2 infection in hospitalized individuals, with no significant difference in performance between tests and consistently high sensitivity > 100 days after symptom onset (Supplemental Table 2). However, for nonhospitalized individuals, the sensitivity of the TCR-based test was significantly higher than serology (Table 2). Notably, in nonhospitalized individuals, wider differences in sensitivity were seen for samples tested > 100 days from symptom onset (86.2%, 76.6%, and 72.3% for the T cell test, EUROIMMUN, and Abbott ARCHITECT, respectively; Table 2). Together, these results suggest that the rate of signal may decline faster for antibody-based tests relative to T cell testing, particularly in nonhospitalized individuals (Figure 5, C and D, and Supplemental Figure 3), consistent with findings that some individuals undergo loss of detectable antibodies or seroreversion (27, 28).

### SARS-CoV-2–specific T cell responses were detectable in a subset of convalescent cases negative by nAb and serology testing.

**Figure 6:**
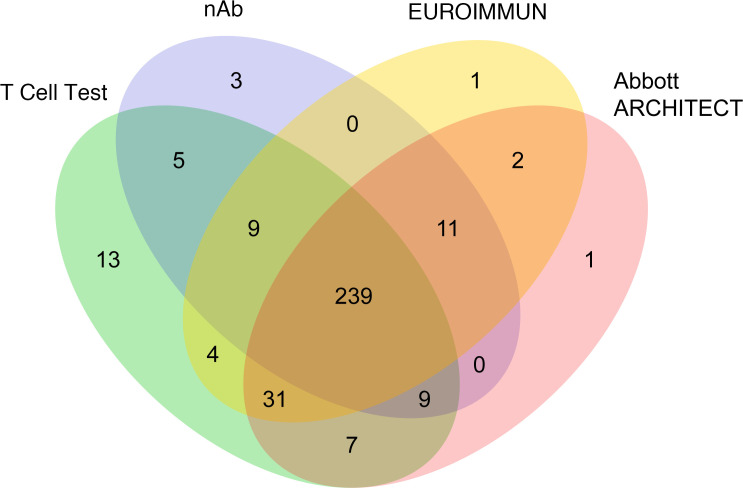
Concordance among T cell, EUROIMMUN, Abbott ARCHITECT, and nAb assays. Schematic showing overlapping SARS-CoV-2^+^ test results obtained using the T cell (green), EUROIMMUN (yellow), Abbott ARCHITECT (pink), and nAb (blue) assays (*n* = 357).

Comparison of T cell and serology testing revealed discordant results for a subset of samples (Figure 5, E and F; Figure 6; and Table 3). The EUROIMMUN, Abbott ARCHITECT, and nAb tests each identified 14 samples as SARS-CoV-2^+^ that were classified as SARS-CoV-2^–^ by the T cell assay, while the T cell assay identified 34, 31, and 55 positive samples that were classified as negative or undetectable by the EUROIMMUN, Abbott ARCHITECT, and nAb assays, respectively. Notably, 53 of the 55 T cell assay–positive samples with undetectable nAb titers (<1:40) originated from nonhospitalized cases. T cell testing also identified 13 positive samples among the 35 samples classified as negative by all 3 antibody assays, all originating from nonhospitalized individuals (Figure 5, E and F; Figure 6; and Supplemental Table 2). Of the 22 samples without detectable immune responses on any test, only 1 was from a hospitalized case. The remainder originated from individuals with mild disease and a lower incidence of fever compared with other cases (*P* = 0.01, 2-tailed *t* test). These results are consistent with published reports stating that some individuals, particularly those with mild disease, generate a T cell response to SARS-CoV-2 in the absence of detectable antibodies (27, 29, 30).

## Discussion

In this study, we have applied TCR immunosequencing to samples collected from a convalescent blood donor program in order to characterize the breadth/depth and kinetics of the T cell response to SARS-CoV-2 and evaluate correlations with clinical variables. This study builds on our previously reported data (17, 18, 23) by using MIRA to define the antigen specificity and HLA restrictions of SARS-CoV-2–associated TCRs and identify T cell subpopulations that best correlate with nAb titers. In addition, the extensive clinical metadata available for this cohort have allowed a detailed comparison of the strength and duration of T cell and humoral responses to SARS-CoV-2 in patients with varying disease severity. Results from this study provide evidence for robust, persistent T cell responses to SARS-CoV-2 infection, particularly in patients with more severe disease requiring hospitalization, but also in a subset of individuals with no antibody responses on either nAb or serologic assays. Our results contribute to our understanding of the T cell response to SARS-CoV-2 and underscore the importance of understanding T cell–mediated immunity in addition to serologic immunity for informing the response to the COVID-19 pandemic.

Results from this study demonstrate that the depth and breadth of the T cell response are positively correlated with nAb titers. By leveraging MIRA-based TCR assignments, we show that class II–associated TCRs for spike protein and nucleocapsid phosphoprotein likely form the basis for positive correlations with nAb titers, as no significant correlations were observed for TCRs assigned to other antigens or to CD8^+^ T cells. Therefore, although most nAbs are presumed to target the spike protein (15), our results suggest that CD4^+^ cells responding to antigens from spike and other viral proteins may support the development of functional humoral immunity. In the present study, we demonstrated that at least a portion of the TCRs assigned to CD4^+^ SARS-CoV-2–specific T cells using our enhanced sequence and MIRA methodologies are also restimulated by bona fide SARS-CoV-2 viral antigen. These results provide orthogonal validation of our TCR discovery approach, although additional studies are needed to fully validate the functional status of the SARS-CoV-2–associated TCRs in the classifier.

CD4^+^ T cells have also been suggested to serve as a coordinator of cellular and humoral immunity in vaccinated individuals, as levels of CD4^+^ T cells after the first dose of a COVID-19 mRNA vaccine were predictive of post-second-dose humoral and CD8^+^ T cell responses (31). Analysis of the T cell response is also relevant for SARS-CoV-2 vaccination strategies (32), and recently updated FDA guidance recommends evaluation of vaccine immunogenicity endpoints via nAb response, as well as exploration of the potential impact of emerging viral variants on vaccine-induced immunity (33). We have recently developed a TCR classifier capable of discriminating between vaccine-mediated and infection-mediated T cell responses (34) that could provide insight into the role of T cell responses in supporting COVID-19 in vaccinated individuals and those with hybrid immunity. From our own experiments mapping TCRs to specific viral antigens (17), as well as other reports (9), we expect that most T cell responses will not be substantially affected by viral strain variations, as the majority of antigens do not overlap with sites of variation. Recent analyses showing that T cell epitopes are largely conserved in the highly mutated SARS-CoV-2 Omicron variant are consistent with this hypothesis (35, 36).

In addition to nAb titers, we found that T cell responses are significantly correlated with clinical measures of disease severity, including hospitalization, fever, and difficulty breathing. T cell responses were greater in men and older adults, consistent with increased rates of severe illness and hospitalization in these groups (26). The association between magnitude of the T cell response and clinical indicators of disease severity is consistent with reports showing heightened T cell responses in symptomatic or hospitalized individuals that can persist months after infection (3, 23). Importantly, individuals who experience more severe disease may also exhibit improved protection from emerging viral variants, as individuals hospitalized due to COVID-19 exhibit a greater binding and neutralization capacity against SARS-CoV-2 variants of concern compared with individuals who experienced a mild infection (37). We hypothesize that increased viral load, longer viral persistence, and/or higher levels of immune activation during acute SARS-CoV-2 infection may partly underlie the association between more severe COVID-19 illness and greater activation and durability of the T cell response.

Evaluation of samples collected from individuals up to 190 days after symptom onset revealed that TCR testing has high sensitivity for indicating diagnosis of prior SARS-CoV-2 infection up to at least 6 months after infection. We have previously described the clinical validation and performance of a TCR-based assay (T-Detect COVID, a commercially available test) for diagnosing past SARS-CoV-2 infection with ~100% specificity; high sensitivity, equivalent to or higher than that of commercial serologic testing; and lack of pathogen cross-reactivity (18). In the present study, using an updated classifier (see Methods), performance of the TCR-based test was equivalent to serology testing in hospitalized individuals and was more sensitive than serology in nonhospitalized individuals, particularly > 100 days from initial symptom onset. The greatest difference in performance was observed at > 150 days, but the sample size was limited over these ranges, and additional research is needed to confirm performance compared with these and other serology tests currently in use, such as the Roche Elecsys Anti–SARS-CoV-2 assay. As only a minority of individuals with COVID-19 require hospitalization (5.3%, derived from CDC data for US cases; ref. 38), the improved performance observed in symptomatic, nonhospitalized individuals supports the utility of T cell–based testing for monitoring past infection in the real world, where the vast majority of infections occur in the outpatient setting. Furthermore, in alignment with previously reported results (23), we demonstrate that T cell testing can identify SARS-CoV-2 responses in a high proportion of individuals with no detectable antibody responses on either nAb or serologic assays. This observation underscores the necessity of measuring both cellular (CD4^+^/CD8^+^ T cells) and humoral immunity for thorough assessment of the immune response to SARS-CoV-2 infection and suggests that T cell testing provides important additive diagnostic value.

The implications of this study are limited by a lack of diversity in the participant cohort. As would be expected in a population of convalescent plasma donors, the demographics of the study population skewed older. A previous study has shown 1.16- to 1.24-fold higher T cell responses by decade of age (39), and another study that compared T cell responses in adults and children found that the magnitude of T cell responses were lower in pediatric cases (40). Given that the severity of COVID-19 increases with age (41) and that we have shown that depth and breadth of the T cell response measured by this assay increases with disease severity, it is possible that diagnostic sensitivity could be lower in younger individuals. However, an analysis of the representation of public SARS-CoV-2–specific TCRs has suggested that representation of these TCRs is higher among individuals < 60 years of age compared with those older than 60 years of age (42). Based on these divergent data, additional analyses are needed to assess the sensitivity of the assay in younger and asymptomatic individuals. A comparison of public and private T cell responses across these demographics could also provide insight into whether assays evaluating public TCR responses are reflective of the private component across the population. In addition to age, the study population also had limited racial and ethnic diversity. Although the training set used for development of the SARS-CoV-2 TCR classifier included 40 Hispanic and 67 Black individuals positive for SARS-CoV-2 among the 784 cases, further studies are needed to understand the sensitivity of the assay across diverse racial and ethnic groups. Because HLA alleles influence TCR sequences and are unequally distributed worldwide (43, 44), both training data sets and test cohorts should ideally include greater diversity.

Beyond the demographics of the cohort, the present study was also limited by restriction of the cohort to symptomatic cases. Recent analyses of SARS-CoV-2–specific T cell responses by ELISpot and/or cytokine assays have shown that the magnitude of the T cell response ranges from no difference to up to 50% greater in symptomatic cases compared with asymptomatic cases (40, 45, 46); thus, it is possible that sensitivity of TCR testing may be lower in individuals with asymptomatic disease. Despite these limitations, our results suggest that analysis of the TCR repertoire from small-volume blood samples may be useful as a modality for evaluating past infection and immune protection months after COVID-19 illness. T cell–based testing may overcome several challenges with nAb and serologic testing, including labor intensity, biohazard risks, scalability, incomplete or absent antibody signal in nonsevere illness, and limited antibody persistence, although universal access to this diagnostic technology may be limited by expense and a lack of local laboratory availability (47). In addition, T cell–based testing may have clinical utility in individuals unlikely to develop an antibody response, such as those taking anti-CD20 therapies (32). Future studies encompassing diverse populations and longer-term follow-up are needed to better define the nature and duration of the detectable T cell response and its utility as a biomarker for assessing natural and vaccine-mediated immunity.

## Methods

### Clinical sample collection.

The Virology Research Clinic at the University of Washington began enrollment in an IRB-approved study in April 2020 (NCT04338360). The study recruited individuals with a laboratory-confirmed SARS-CoV-2 infection who volunteered to be considered for convalescent plasma donation. Patient characteristics are provided in Table 1; age, race, and sex were self-reported.

### Immunosequencing of TCR repertoires.

Genomic DNA was extracted from cryopreserved PBMCs. As much as 18 μg of input DNA was then used to perform immunosequencing of CDR3 from TCR-β chains using the immunoSEQ assay (Adaptive Biotechnologies). Briefly, input DNA was amplified in a bias-controlled multiplex PCR, followed by high-throughput sequencing. Sequences were collapsed and filtered to identify and quantitate the absolute abundance of each unique TCR-β CDR3 region amino acid sequence for further analysis, as previously described (48–50).

### T-Detect model to characterize the T cell response to SARS-CoV-2.

Classification of prior SARS-CoV-2 infection, as well as the clonal depth and breadth of the T cell response, were determined using methods published by Snyder et al. (17). Briefly, TCR repertoires from 784 unique cases of reverse transcription PCR–confirmed (RT-PCR–confirmed) SARS-CoV-2 infection and 2447 healthy controls collected before 2020 were compared using 1-tailed Fisher’s exact tests to identify 4469 public TCR-β amino acid sequences (enhanced sequences) significantly enriched in SARS-CoV-2^+^ samples. None of the samples used for model training were from the clinical cohort investigated in the present study.

Following initial selection of enhanced sequences, some filtering of the sequence list was performed to remove potential false positives. Specifically, we reasoned that TCRs associated with CMV seropositivity or HLA alleles in non–COVID-19 healthy populations were unlikely to be specific to SARS-CoV-2. We therefore identified TCRs associated with CMV seropositivity or any of over 100 HLA-I or HLA-II subtypes using a 1-tailed Fisher’s exact test applied to TCR-β repertoires of ~2000 healthy controls with available HLA genotyping and CMV serotyping data. A total of 182 candidate SARS-CoV-2–associated TCR sequences were also associated with an HLA subtype or CMV seropositivity and were removed, leaving 4287 enhanced sequences.

The final list of enhanced sequences was used to develop a classifier predicting recent or past infection with SARS-CoV-2 using a simple 2-feature logistic regression, with independent variables E and N, where E is the number of unique TCR-β DNA sequences that encode an enhanced sequence and N is the total number of unique productive TCR-β DNA sequences in that subject. We define the T cell test score to be the log-odds of the probability of this logistic regression model. A decision boundary on this T cell test score representing 99.8% specificity on 1657 controls was used to define the test-positive threshold used in the present study; this model was identical to that used in another recent analysis (23).

The narrowed list of enhanced sequences was also used to calculate the clonal depth and breadth using the same formulae described in more detail by Snyder et al. (17). Briefly, treating unique TCR DNA sequences observed in a repertoire as distinct clonotypes, clonal breadth represents the fraction of all observed clonotypes in a repertoire that represent TCRs associated with SARS-CoV-2. Clonal depth accounts for the extent of clonal expansion of each SARS-CoV-2–specific clonotype. With *t_i_* representing the total number of T cells observed for clonotype I, *N* representing the total number of T cells in a sequenced repertoire, and *D* representing the set of all disease-associated clonotypes, we estimate the clonal generations for each clonotype as log_2_ (1 + *t_i_*). Clonal depth, normalizing for depth of sampling, is calculated as the following: ∑_i∈d_log_2_ (1 + *t_i_*) – log_2_ (*N*).

### Estimating the SARS-CoV-2 protein antigen-specific T cell response.

Public TCRs were assigned to SARS-CoV-2 antigens by cross-referencing enhanced sequences identified via our case/control design with TCRs observed in multiplexed antigen-stimulation experiments, both described in prior work (17). To maximize the number of TCR-antigen assignments, we identified a set of public TCRs from an augmented sample of repertoire data comprising prior training and validation repertoires, 1143 additional SARS-CoV-2^+^ samples, and more than 1800 samples identified as SARS-CoV-2^–^ from another large study (23). The final sample of repertoires, consisting of 1927 cases and 4135 controls, was used to identify ~500,000 candidate public SARS-CoV-2–specific TCRs with *P* < 0.05 (Fisher’s exact test). We cross-referenced this list of TCRs with a set of ~400,000 TCRs independently derived from multiplexed antigen-stimulation experiments to yield 3381 overlapping TCRs in both data sets with protein and CD4^+^/CD8^+^ assignments determined based on antigen-stimulation experiments.

### Functional validation of enhanced sequences.

AIM-based enrichment of SARS-CoV-2–specific T cells was performed using PBMCs from 2 independent convalescent samples from each of 3 convalescent individuals from the original study population. Samples from these 3 individuals (patients W001, W005, and W012) were selected based on their inclusion in a data set used for T cell epitope discovery in a separate study (51). Each of these 3 patients required hospitalization and intensive care admission for treatment of COVID-19. PBMCs were stimulated with whole, ultraviolet light–killed, cell-associated SARS-CoV-2 with inclusion of autologous monocyte-derived DCs (moDC), as recently described (51, 52). In brief, SARS-CoV-2 strain WA1 was expanded in Vero-E6 cells transfected with angiotensin-converting enzyme-2 (*ACE2*) and transmembrane serine protease 2 (*TMPRSS2*; gifted by Michael Diamond, Washington University, St. Louis, Missouri, USA). Antigen created by freeze-thaw cycles was documented as inactivated by plaque assay. Antigen was added to autologous moDC for 5 hours, followed by culture with autologous PBMC for 18 hours. Cells were stained for viability with 7-actinomycin D (Thermo Fisher Scientific) and with the following fluorochrome-conjugated monoclonal antibodies: CD3-PE (clone UCHT-1, BioLegend); CD4-APC-Cy7 (clone RPA-T4, BD Biosciences); CD8-FITC (clone 3B5, Thermo Fisher Scientific); CD137-APC (clone 4B4-1, BD Biosciences); and CD69-BV421 (clone FN50, BioLegend). Live CD3^+^CD4^+^CD8^–^CD137^+^CD69^+^ cells were sorted using BD FacsAria II (Department of Immunology Cell Analysis Facility, University of Washington). DNA was extracted with a Qiagen DNA Micro kit for immunosequencing of TCR repertoires performed, as described above.

### Serology assays.

Methods used for serologic assays to assess antibody responses have been described in detail previously (24). Plasma was isolated within 12 hours of collection prior to serologic testing with the EUROIMMUN or Abbott ARCHITECT assays. The EUROIMMUN anti–SARS-CoV-2 IgG ELISA was performed according to the manufacturer’s protocol. The cutoff for positivity was an OD ratio ≥ 1.1 based on the manufacturer-provided reference. The Abbott SARS-CoV-2 IgG chemiluminescent microparticle immunoassay (Abbott ARCHITECT) was performed according to the manufacturer’s instructions. A kit calibrator was used to generate the assay index, with values ≥ 1.4 considered positive. nAb titers were measured in plasma that was frozen within a few hours of specimen collection, shipped on dry ice, and then thawed at the time of the assay. nAb titers were assessed using a fluorescence reduction neutralization assay assessing inhibition of SARS-CoV-2 replication in Vero E6 cells (CRL-1586, ATCC). SARS-CoV-2 (2019-nCoV/USA-WA1-A12/2020, US CDC) was detected using a primary antibody targeting nucleoprotein (40143-MM05, Sino Biological) and an Alexa Fluor 594–conjugated secondary antibody (A-21125, Invitrogen). Plasma dilutions of 1:40 that did not result in at least a 50% reduction in viral titer are reported as undetectable (<1:40), as previously described (53).

### Data availability.

T cell repertoire profiles and antigen annotation data from multiplexed antigen-stimulation experiments are available through the ImmuneCODE resource (54) and can be downloaded from the Adaptive Biotechnologies immuneACCESS site under the immuneACCESS Terms of Use at https://clients.adaptivebiotech.com/pub/elyanow-2022-jci (DOI: 10.21417/RE2022JCI). AIM sequencing data are available upon request at https://www.adaptivebiotech.com/medical-information/

### Statistics.

The Pingouin package in Python (55) was used to calculate Spearman’s rank correlations between antibody titers and CD4^+^ and CD8^+^ T cell responses and report the 2-sided significance. To disentangle confounding correlations, partial Spearman’s rank correlations were calculated between spike, nucleocapsid phosphoprotein, and other antigen-specific T cell responses and antibody titers; 2-sided significance was reported. Partial correlation coefficients and *P* values are denoted with tildes when included in figures. *P* values less than 0.05 were considered significant. When calculating partial correlations between antibody titers and specific categories of T cell responses (CD4^+^ T cell response specific to spike, nucleocapsid phosphoprotein, and all other assayed proteins), the other T cell groups served as covariates. These partial correlations describe the correlation between 2 variables that cannot be explained by the covariates, representing a conservative measure of correlation.

Significant associations between T cell clonal breadth and clinical variables were assessed by the Mann-Whitney *U* test in univariate analysis and by multivariable linear regression with age, sex, hospitalization, fever, difficulty breathing, and TCR rearrangements as variables. Mid-P McNemar’s test was used to compare the sensitivities of the T cell test and commercial serological assays. *P* values less than 0.05 were considered significant.

### Study approval.

This study was approved by the University of Washington IRB (STUDY00004312). All participants provided written informed consent.

## Author contributions

DMK and AW designed the initial study. This follow-up investigation was conceived by DMK and AW with TMS, SCD, IMK, LB, and HSR. Data analysis was performed by RE, TMS, and RMG with support from JB, AW, SS, MHW, CM, ALG, MRH, DHM, and DMK. LJ, TYH, and MG conducted AIM using whole SARS-CoV-2 antigen. RE, TMS, SCD, RMG, DHM, LB, TM, HSR, and DMK contributed to data interpretation. RE, TMS, SCD, RMG, and DMK wrote the manuscript, with contributions from JB, AW, SS, MHW, CM, ALG, MRH, IMK, HJZ, JMC, LB, TM, and HSR. RE and TMS share the first-author position and are listed in alphabetical order. All authors approved of the final manuscript.

## Supplementary Material

Supplemental data

ICMJE disclosure forms

## Figures and Tables

**Table 3:**
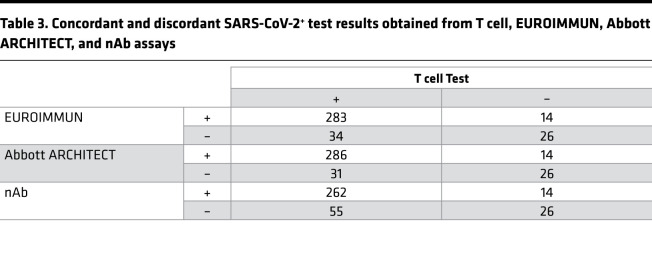
Concordant and discordant SARS-CoV-2^+^ test results obtained from T cell, EUROIMMUN, Abbott ARCHITECT, and nAb assays

**Table 1:**
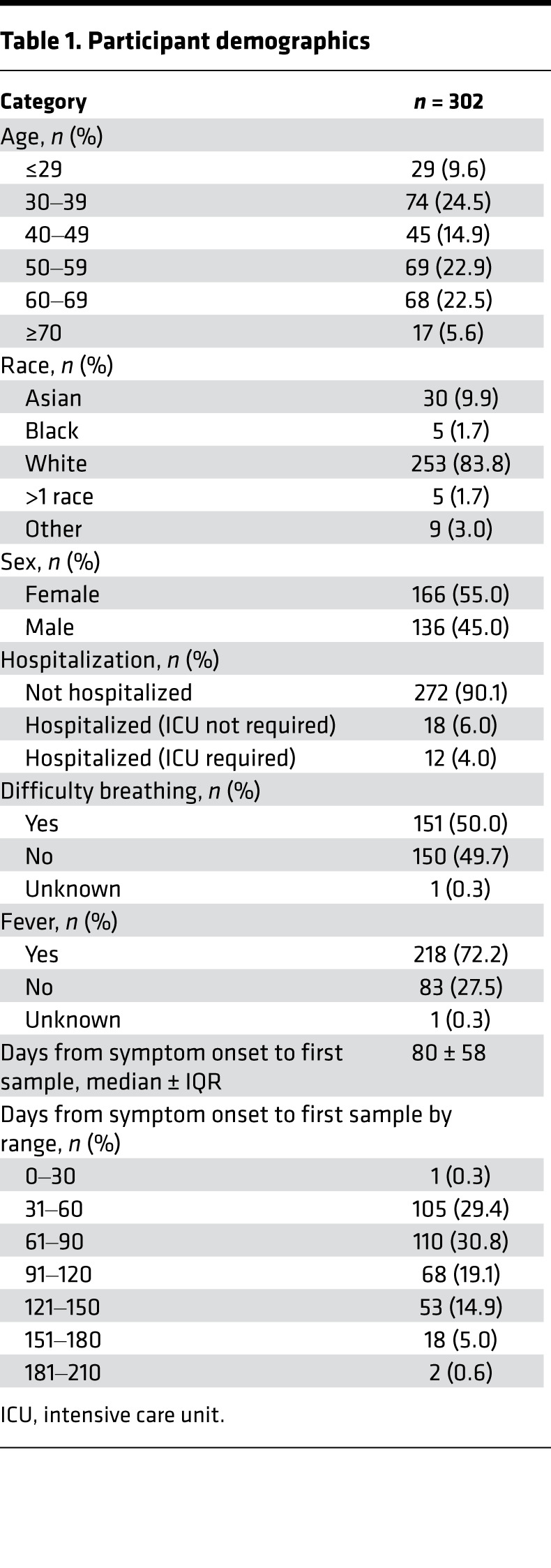
Participant demographics

**Table 2:**
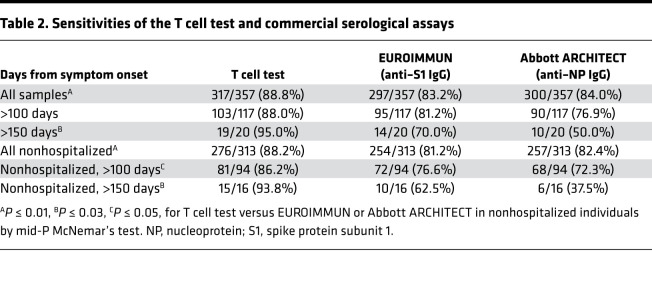
Sensitivities of the T cell test and commercial serological assays

## Version 1 04/19/2022

In-Press Preview

## Version 2 05/23/2022

Electronic publication
