# Supplementary material for: T cell receptor sequencing identifies prior SARS-CoV-2 infection and correlates with neutralizing antibodies and disease severity
Source: JCI Insight. 2022 May 23;7(10):e150070. doi: 10.1172/jci.insight.150070 (PMC9220924; doi:10.1172/jci.insight.150070)
Supplement: Supplemental data [file jciinsight-7-150070-s039.pdf]

T-cell receptor sequencing identifies prior SARS-CoV-2 infection and correlates with neutralizing antibodies and disease severity

Rebecca Elyanow,[1*] Thomas M. Snyder,[1*] Sudeb C. Dalai,[1,2**] Rachel M. Gittelman,[1] Jim Boonyaratanakornkit,[3,4] Anna Wald,[3,4,5,6] Stacy Selke,[6] Mark H. Wener,[3,6] Chihiro Morishima,[6] Alexander L. Greninger,[6] Michael Gale Jr.,[7,8,9] Tien-Ying Hsiang,[7] Lichen Jing,[3] Michael R. Holbrook,[10] Ian M. Kaplan,[1] H. Jabran Zahid,[11] Damon H. May,[1] Jonathan M. Carlson,[11] Lance Baldo,[1] Thomas Manley,[1**] Harlan S. Robins,[1] David M. Koelle[3,4,6,9,12]

*These authors share the first-author position and are listed in alphabetical order.

**Author was an Adaptive employee at time of research.

[1]Adaptive Biotechnologies, Seattle, Washington, USA

[2]Stanford University School of Medicine, Stanford, California, USA

[3]Department of Medicine, University of Washington, Seattle, Washington, USA

[4]Fred Hutchinson Cancer Research Center, Seattle, Washington, USA

[5]Department of Epidemiology, University of Washington, Seattle, Washington, USA

[6]Department of Laboratory Medicine and Pathology, University of Washington, Seattle, Washington, USA

[7]Department of Immunology, University of Washington, Seattle, Washington, USA

[8]Department of Microbiology, University of Washington, Seattle, Washington, USA

[9]Department of Global Health, University of Washington, Seattle, Washington, USA

[10]National Institute of Allergy and Infectious Diseases Integrated Research Facility, Frederick, Maryland, USA

[11]Microsoft Research, Redmond, Washington, USA

[12]Benaroya Research Institute, Seattle, Washington, USA

Corresponding author:

David M. Koelle, MD

750 Republican Street

Room E651, Mail Stop 358061

Seattle, WA 98109 USA

Tel (206) 616-1940

DKoelle@medicine.washington.edu

Supplemental Materials

Supplemental Tables

Supplemental Table 1. Longitudinal sensitivity of the T-Cell, EUROIMMUN, and Abbott ARCHITECT tests by time from symptom onset.

Days From Symptom Onset	T-Cell Test		EUROIMMUN (anti-S1 IgG)		Abbott ARCHITECT (anti-NP IgG)	
	Sensitivity	95% CI	Sensitivity	95% CI	Sensitivity	95% CI
**All samples[A]**	317/357 (88.8%)	85.1–91.9	297/357 (83.2%)	79.0–86.8	300/357 (84.0%)	79.8–87.7
**29–50**	60/70 (85.7%)	77.1–92.9	57/70 (81.4%)	71.4–90.0	59/70 (84.3%)	75.7–91.4
**51–100**	154/170 (90.6%)	85.9–94.7	145/170 (85.3%)	80.0–90.6	146/170 (88.8%)	83.5–93.5
**101–150**	84/97 (86.6%)	79.4–92.8	81/97 (83.5%)	76.3–90.7	80/97 (82.5%)	75.3–89.7
**150–190[B]**	19/20 (95%)	85.0–100	14/20 (70%)	50.0–90.0	10/20 (50%)	30.0–70.0

[A] P=0.01, T-cell test vs EUROIMMUN or Abbott ARCHITECT by mid-p McNemar's test.
[B] P<0.03, T-cell test vs EUROIMMUN or Abbott ARCHITECT by mid-p McNemar's test.
NP, nucleoprotein; S1, spike protein subunit 1.

Supplemental Table 2. Longitudinal sensitivity of the T-cell, EUROIMMUN, and Abbott ARCHITECT tests for hospitalized and non-hospitalized patients.

Days From Symptom Onset	T-Cell Test		EUROIMMUN (anti-S1 IgG)		Abbott ARCHITECT (anti-NP IgG)	
	Hospitalized	Non-Hospitalized	Hospitalized	Non-Hospitalized	Hospitalized	Non-Hospitalized
**All samples[A]**	41/44 (93.2%)	276/313 (88.2%)	44/44 (97.7%)	254/313 (81.2%)	42/45 (95.5%)	257/313 (82.4%)
**29–50**	3/4 (75%)	57/66 (86.4%)	4/4 (100%)	53/66 (80.3%)	4/4 (100%)	55/66 (83.3%)
**51–100**	16/17 (94.1%)	138/153 (90.2%)	16/17 (94.1%)	129/153 (84.3%)	16/17 (94.1%)	135/153 (88.2%)
**101–150**	18/19 (94.7%)	66/78 (84.6%)	19/19 (100%)	62/78 (79.5%)	18/19 (94.7%)	62/78 (79.5%)
**150–190**	4/4 (100%)	15/16 (93.8%)	4/4 (100%)	10/16 (62.5%)	4/4 (100%)	6/16 (37.5%)

[A] P=0.15 for hospitalized individuals and P=0.01 for non-hospitalized individuals comparing T-cell test vs EUROIMMUN or Abbott ARCHITECT by mid-p McNemar's test.

NP, nucleoprotein; S1, spike protein subunit 1.

Supplemental Figures

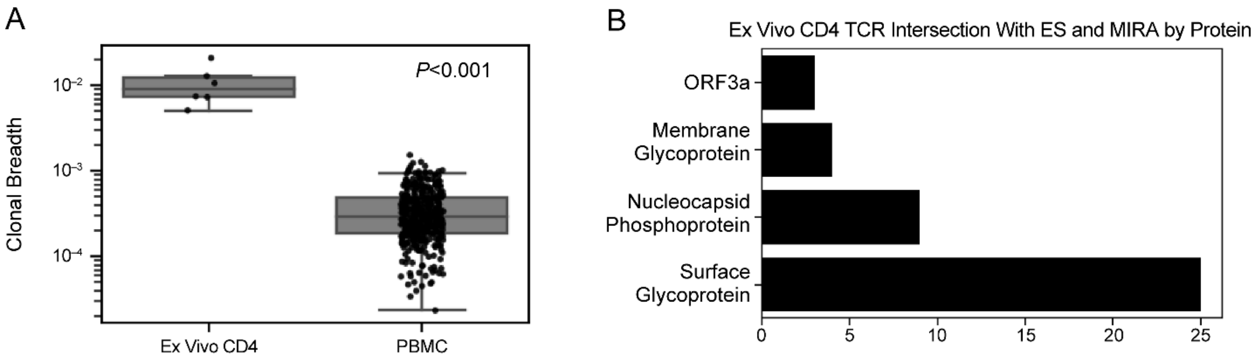

Supplemental Figure 1. Quantification of SARS-CoV-2–specific enhanced sequences and MIRA TCRs in ex vivo AIM-sorted CD4+ T cells. (A) PBMCs from 3 convalescent donors at 2 timepoints were subjected to AIM-based enrichment of SARS-CoV-2–specific T-cells using whole cell-associated viral antigen. Clonal breadth of SARS-CoV-2–specific enhanced sequences was compared between AIM-sorted CD4+ T cells and matched convalescent PBMCs. Significance was evaluated by Mann-Whitney U test. Data are expressed as median ± interquartile ranges. (**B**) Intersection of SARS-CoV-2–specific TCRs present in ex vivo AIM-sorted CD4+ T-cells and MIRA TCR set, annotated by protein specificity.

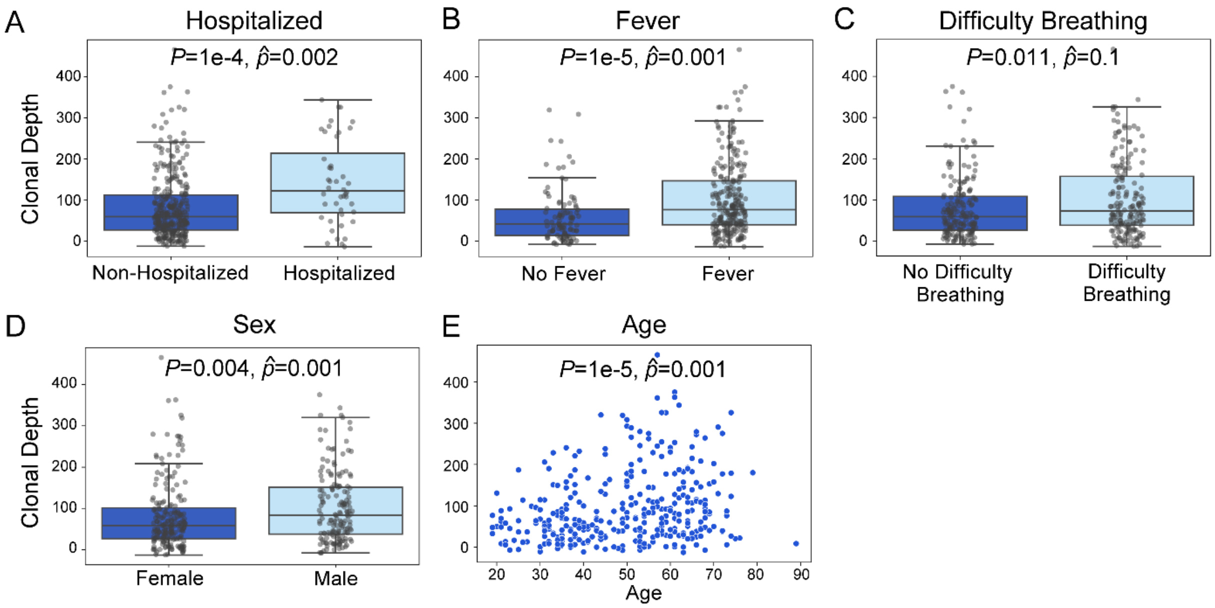

Supplemental Figure 2. Association of T-cell clonal depth with clinical variables. Correlation of clonal depth with (**A**) hospitalization, (**B**) fever, (**C**) difficulty breathing, (**D**) sex, and (**E**) age was evaluated by univariate Mann-Whitney U test (p) and multivariate linear regression with age, sex, hospitalization, fever, difficulty breathing, and TCR rearrangements as variables ($\hat{p}$). Data are expressed as median ± interquartile ranges (*n*=302). Population means and 95% confidence interval (CI) values for panels A–E were as follows: (**A**) Non-hospitalized, 88.3 (97.0-97.8), hospitalized, 168.3 (126.2-210.5); (**B**) no fever, 63.6 (50.3-76.9, fever, 108.2 (96.3-120.1); (**C**) no difficulty breathing, 85.6 (73.4-97.8), difficulty breathing, 105.4 (90.5-120.2); (**D**) female, 85.0 (72.4-97.6), male, 107.6 (93.1–122.1).

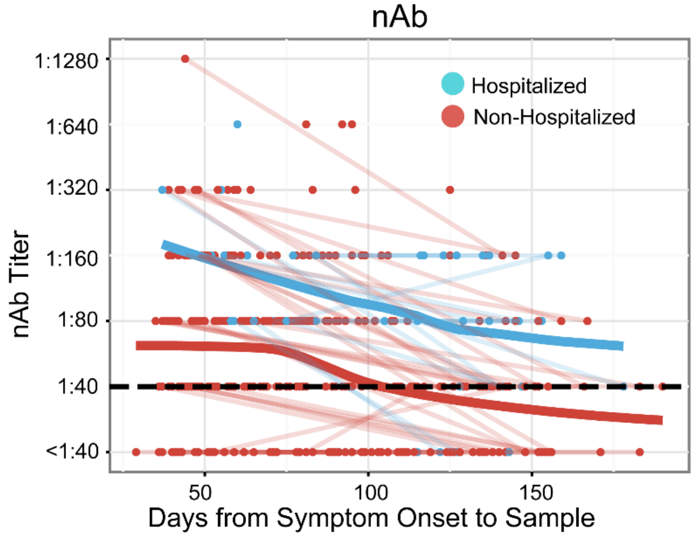

Supplemental Figure 3. Neutralizing antibody titer as a function of days from symptom onset to sample collection. Blue and red dots indicate hospitalized and non-hospitalized individuals, respectively. Trend lines connect visit 1 and visit 2 samples from the same individual. Blue and red bold trend lines indicate smoothed means [locally estimated scatterplot smoothing, LOESS (1)] for hospitalized and non-hospitalized individuals (*n*=302), respectively.

Supplemental Reference

1. Cleveland WS. LOWESS: a program for smoothing scatterplots by robust locally weighted regression. *Am Stat.* 1981;35(1):54.
